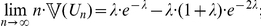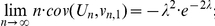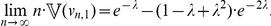# Extrapolation of Urn Models via Poissonization: Accurate Measurements of the Microbial Unknown

**DOI:** 10.1371/journal.pone.0021105

**Published:** 2011-06-28

**Authors:** Manuel E. Lladser, Raúl Gouet, Jens Reeder

**Affiliations:** 1 Department of Applied Mathematics, University of Colorado, Boulder, Colorado, United States of America; 2 Centro de Modelamiento Matemático (CNRS UMI 2807), Universidad de Chile, Santiago, Chile; 3 Department of Chemistry and Biochemistry, University of Colorado, Boulder, Colorado, United States of America; University of New Orleans, United States of America

## Abstract

The availability of high-throughput parallel methods for sequencing microbial communities is increasing our knowledge of the microbial world at an unprecedented rate. Though most attention has focused on determining lower-bounds on the 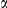-diversity i.e. the total number of different species present in the environment, tight bounds on this quantity may be highly uncertain because a small fraction of the environment could be composed of a vast number of different species. To better assess what remains unknown, we propose instead to predict the fraction of the environment that belongs to unsampled classes. Modeling samples as draws with replacement of colored balls from an urn with an unknown composition, and under the sole assumption that there are still undiscovered species, we show that conditionally unbiased predictors and exact prediction intervals (of constant length in logarithmic scale) are possible for the fraction of the environment that belongs to unsampled classes. Our predictions are based on a Poissonization argument, which we have implemented in what we call the Embedding algorithm. In fixed i.e. non-randomized sample sizes, the algorithm leads to very accurate predictions on a sub-sample of the original sample. We quantify the effect of fixed sample sizes on our prediction intervals and test our methods and others found in the literature against simulated environments, which we devise taking into account datasets from a human-gut and -hand microbiota. Our methodology applies to any dataset that can be conceptualized as a sample with replacement from an urn. In particular, it could be applied, for example, to quantify the proportion of all the unseen solutions to a binding site problem in a random RNA pool, or to reassess the surveillance of a certain terrorist group, predicting the conditional probability that it deploys a new tactic in a next attack.

## Introduction

A fundamental problem in microbial ecology is the “rare biosphere” [1] i.e. the vast number of low-abundance species in any sample. However, because most species in a given sample are rare, estimating their total number i.e. 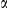-diversity is a difficult task [2], [3], and of dubious utility [4], [5]. Although parametric and non-parametric methods for species estimation show some promise [6], [7], microbial communities may not yet have been sufficiently deeply sampled [8] to test the suitability of the models or fit their parameters. For instance, human-skin communities demonstrate an unprecedented diversity within and across skin locations of same individuals, with marked differences between specimens [9].

In an environment composed of various but an unknown number of species, let 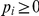 be the proportion in which a certain species 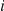 occurs. Samples from microbial communities may be conceptualized as sampling–with replacement–different colored balls from an urn. The urn represents the environment where samples are taken: soil, gut, skin, etc. The balls represent the different members of the microbial community, and each color is a uniquely defined operational taxonomic unit.

In the non-parametric setting, the urn is composed by an unknown number of colors occurring in unknown relative proportions. In this setting, the 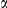-diversity of the urn [10] corresponds to the cardinality of the set 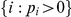. Although various lower-confidence bounds for this parameter have been proposed in the literature [11]–[14], tight lower-bounds on 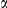-diversity are difficult in the non-parametric setting because a small fraction of the urn could be composed by a vast number of different colors [15]. Motivated by this, we shift our interest to predicting instead the fraction of balls with a color unrepresented in the first 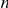 observations from the urn. This is the unobservable random variable:
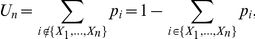
where 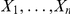 denote the sequence of colors observed when sampling 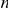 balls from the urn. Notice how 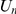 depends both on the specific colors observed in the sample, and the unknown proportions of these colors in the urn. This quantity is very useful to assess what remains unknown in the urn. For instance, the probability of discovering a new color with one additional observation is precisely 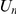, and the mean number of additional observations to discover a new color is 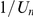. We note that 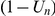 corresponds to what is called the conditional coverage of a sample of size 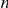 in the literature. For this reason, we refer to 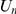 as the *conditional uncovered probability* of the sample.

The expected value of 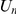 is given by:
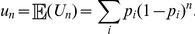
Unlike the conditional uncovered probability of the sample, 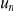 is a parameter that depends on the unknown urn composition but not on the specific colors observed in the sample. Interest in the above quantities or related ones has ranged from estimating the probability distribution of the keys used in the *Kenngruppenbuch* (the Enigma cipher book) in World War II [16], to assessing the confidence that an iterative procedure with a random start has found the global maximum of a given function [17], to predicting the probability of discovering a new gene by sequencing additional clones from a cDNA library [18]. We note that 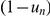 is called the *expected coverage of the sample* in the literature.

Various predictors of 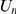 and estimators of 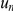 have been proposed in the literature. These are mostly based on a user-defined parameter 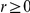 and the statistics 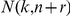, 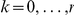; defined as the number of colors observed 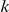-times, when 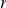 additional balls are sampled from the urn.

**Figure 1:**
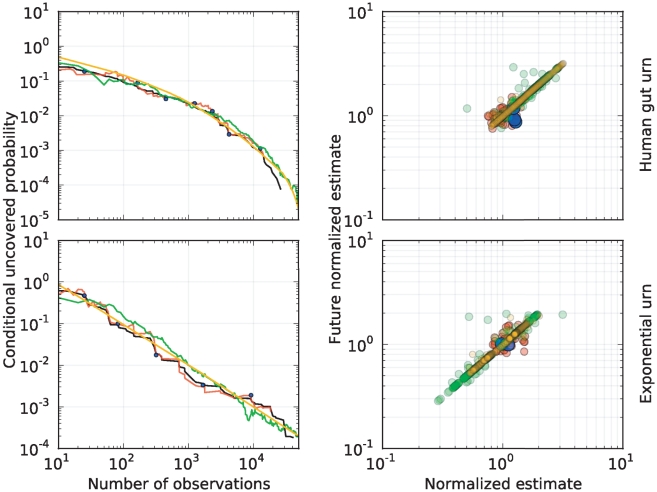
Point predictions in a human-gut and exponential urn. Plots associated with a human-gut (top-row) and exponential urn (bottom-row). Left-column, sequential predictions of the conditional uncovered probability (black), as a function of the number 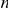 of observations, using Robbins' estimator in equation (1) (green), Starr's estimator in equation (2) (orange), and the Embedding algorithm (blue, red), over a same sample of size 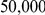 from each urn. Starr's estimator was implemented keeping 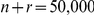. Blue predictions correspond to consecutive outputs of the Embedding algorithm in Table 1, which was reiterated until exhausting the sample using the parameter 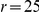. Red predictions correspond to outputs of the algorithm each time a new species was discovered. Right-column, correlation plots associated with consecutive predictions of the conditional uncovered probability (normalized by its true value at the point of prediction), under the various methods. The green and orange clouds correspond to pairs of predictions, 100-observations apart, using Robbins' and Starr's estimators, respectively. Blue and red clouds correspond to pairs of consecutive outputs of the Embedding algorithm, following the same coloring scheme than on the left plots. Notice how the red and blue clouds are centered around 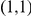, indicating the accuracy of our methodology in a log-scale. Furthermore, the green and orange clouds show a higher level of correlation than the blue and red clouds, indicating that our method recovers more easily from previously offset predictions. In each urn, our predictions used the 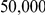 observations and a HPP with intensity one–simulated independently from the urn–to predict sequentially the uncovered probability of the first part of the sample. See Fig. 4 for the associated rank curve in each urn.

Turing and Good [19] proposed to estimate 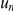 using the biased statistic 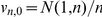. Posteriorly, Robbins [20] proposed to predict 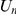 using
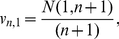
(1)which he showed to be unbiased for 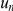 and to satisfy the inequality 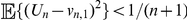. Despite the possibly small quadratic variation distance between 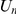 and Robbins' estimator, and as illustrated by the plots on the left side of Fig. 1, when using Robbins' estimator to predict 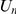 sequentially with 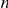 (to assess the quality of the predictions at various depths in the sample), we observe that unusually small or large values of 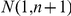 may offset subsequent predictions of 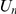. In fact, as seen on the right-hand plots of the same figure, an offset prediction is usually followed by another offset prediction of the same order of magnitude, even 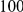 observations later (correlation coefficient of green clouds, 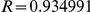 and 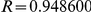 on top- and bottom-right plots).

Subsequently, for each 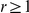, Starr [21] proposed to predict 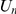 using
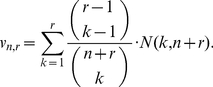
(2)Even though 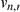 is the minimum variance unbiased estimator of 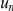 based on 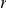 additional observations from the urn [22], Starr showed that 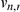 may be strongly negatively correlated with 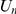 when 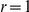 (note that Starr's and Robbins' estimators are identical when 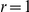). Furthermore, the sequential prediction of 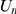 via Starr's estimator is affected by issues similar to Robbins' estimator, which is also illustrated in Fig. 1, even when the parameter 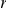 is set as large as possible, namely 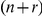 is equal to the sample size (correlation coefficient of orange clouds, 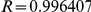 and 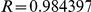 on top- and bottom-right, respectively). We observe that 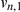 and 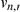 are indistinguishable in a linear scale when 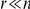 because, for each 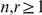, it applies that (see Materials and Methods):
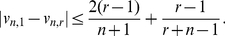
(3)


**Figure 2:**
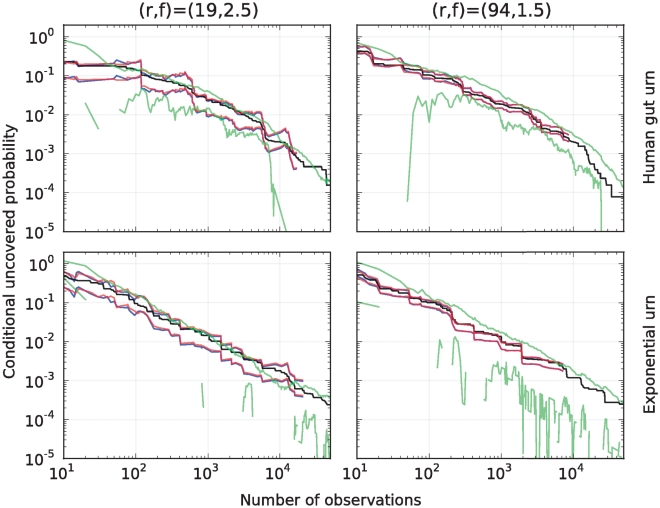
Prediction intervals in the human-gut and exponential urn. 95% prediction intervals for the conditional uncovered probability (black) of the human-gut and exponential urn as a function of the number of observations. Esty's prediction intervals in equation (4) (green), and predictions intervals based on the Embedding algorithm (blue, red), using the parameters 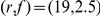 and 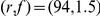 on the left and right, respectively. Blue and red curves correspond to the conservative-lower and -upper prediction intervals for the uncovered probability, respectively. The missing segments on the lower green-curves correspond to Esty's prediction intervals that contained 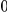. Although the upper- and lower-bound of the Esty's intervals may be of different order of magnitude, our method produces intervals of a constant length in logarithmic scale. This length is controlled by the user-defined parameter 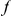. In each urn, our method predicted accurately the uncovered probability of a random sub-sample of the 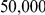 observations from the urn. See Fig. 4 for the associated rank curve in each urn.

In terms of prediction intervals, if 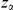 denotes the 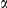 upper quantile of a standard Normal distribution, it follows from Esty's analysis [23] that if 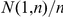 is not very near 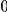 or 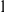 then
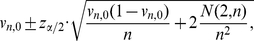
(4)is approximately a 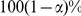 prediction interval for 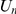. In practice, and as seen in Fig. 2, when the center of the interval is of a similar or lesser order of magnitude than its radius, the ratio between the upper- and lower-bound of these intervals may oscillate erratically, sometimes over several orders of magnitude. This can be an issue in assessing the depth of sampling in rich environments. For instance, to be highly confident that 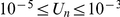 is not of practical use because one may need from 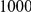 to 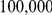 additional observations to discover a new species.

The issues of the aforementioned methods are somewhat expected. On one hand, the problem of predicting 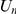 is very different from estimating 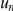: the former requires predicting the exact proportion of balls in the urn with colors outside the random set 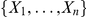, rather than in average over all possible such sets. On the other hand, the point estimators of 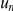 are unlikely to predict 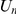 accurately in a logarithmic scale, unless the standard deviation of 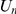 is small relative to 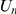. Finally, the methods we have described from the literature were designed for static situations i.e. to predict 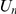 or estimate 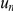 when 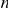 is fixed.

## Results

### Embedding Algorithm

Here we propose a new methodology to address the issues of the methods presented in the Introduction to predict 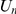. Our methodology lends itself better for a sequential analysis and accurate predictions in a logarithmic scale; in particular, also in a linear scale–though it relies on randomized sample sizes. Due to this, in static situations i.e. for fixed sample sizes, our method only yields predictions for a random sub-sample of the original sample.

Randomized sample sizes are more than just an artifact of our procedure: due to Theorem 1 below, for any predetermined sample size, there is no deterministic algorithm to predict 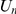 and 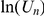 unbiasedly, unless the urn is composed by a known and flat distribution of colors. See the Materials and Methods section for the proofs of our theorems.

**Theorem 1** *If* 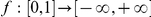 *is a continuous and one-to-one function then the following two statements are equivalent: (i) there is a non-randomized algorithm based on* 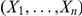 *to predict* 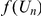 *conditionally unbiased; (ii) the urn is composed by a known and equidistributed number of colors.*

Our methodology is based on a so called Poissonization argument [24]. This technique is often used in allocation problems to remove correlations [25]. It was applied in [26] to show that the cardinality of the random set 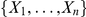 is asymptotically Gaussian after the appropriate renormalization. Mao and Lindsay [27] used implicitly a Poissonization argument to argue that intervals such as in equation (4) have a 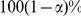 asymptotic confidence, under the hypothesis that the times at which each color in the urn is observed obey a homogeneous Poisson point process (HPP) with a random intensity. Here, asymptotic means that the 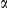-diversity tends to infinity, which entails adding colors into the urn. Our approach, however, is not based on any assumption on the times the data was collected, nor on an asymptotic rescaling of the problem, but rather on the embedding of a sample from an urn into a HPP with intensity 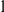 in the semi-infinite interval 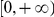. We emphasize that the HPP is a mathematical artifice simulated independently from the urn.

**Figure 3:**
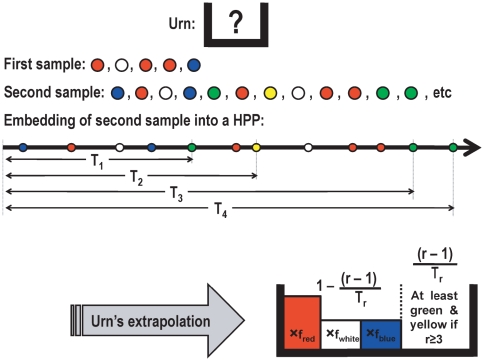
Schematic description of the Embedding algorithm. Suppose that in a first sample from an urn you only observe the colors red, white and blue; in particular, 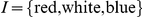. Let 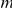 be the unknown proportion in the urn of balls colored with any of these colors i.e. 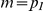. To estimate 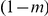, sample additional balls from the urn until observing 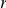 balls with colors outside 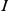. Embed the colors of this second sample into a homogeneous Poisson point process with intensity one; in particular, the average separation of consecutive points with colors outside 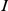 are independent exponential random variables with mean 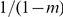. The unknown quantity 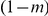 can be now estimated from the random variable 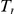. As a byproduct of our methodology, conditional on 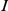, if 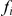 denotes the relative proportion of color 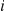 in the first sample then 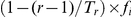 predicts the true proportion of color 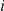 in the urn.

In what follows, 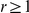 is a user-defined integer parameter. We have implemented the Poissonization argument in what we call the *Embedding algorithm* in Table 1. For a schematic description of the algorithm see Fig. 3 and, for its heuristic, consult the Materials and Methods section.

**Table 1:** Embedding algorithm.

Input:	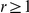 , a set 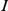 of colors known to be in the urn, and constants 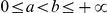 that satisfy condition (5).
Output:	Unbiased predictor of 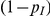 , 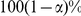 prediction interval for 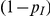 and an updated set 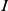 of colors known to belong to the urn.
Step 1.	Assign 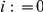 , 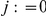 , and 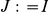 .
Step 2.	While 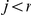 assign 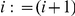 , and sample with replacement a ball from the urn. Let 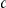 be the color of the sampled ball. If 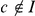 then assign 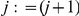 and 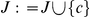 .
Step 3.	Simulate 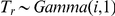 , and assign 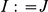 .
Step 4.	Output 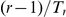 , 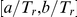 and 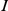 .

Suppose that a set 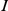 of colors is already known to belong to the urn and let 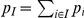 be the coverage probability of the colors in this set. We note that, in the context of the previous discussion, 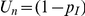 with 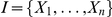.

To predict 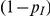, draw balls from the urn until 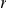 colors outside 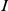 are observed. Visualize each observation as a colored point in the interval 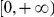. The Poissonization consists in spacing these points out using independent exponential random variables with mean one. Due to the thinning property of Poisson point processes [28], the position 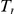 of the point farthest apart from 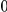 has a Gamma distribution with mean 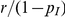. We may exploit this to obtain conditionally unbiased predictors and exact prediction intervals for 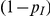 and 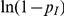 as follows. Regarding direct predictions of 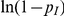, note that measuring 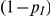 in a logarithmic rather than linear scale makes more sense when deep sampling is possible.

**Theorem 2** *Conditioned on* 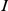 *and the event* 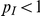 *, the following applies:*

- *If* 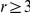 *then* 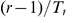 *is unbiased for* 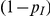, *with variance* 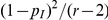.
- *If* 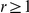 *and* 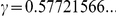 *denotes Euler's constant then* 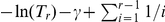 *is unbiased for* 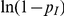, *with variance* 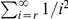, *which is bounded between* 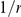 *and* 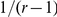.
- *If* 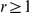, 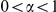 *and* 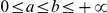 *are such that*

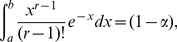
(5)
*then the interval* 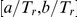 *contains* 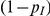 *with exact probability* 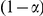; *in particular,* 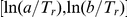 *contains* 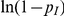 *also with probability* 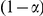.

We note that 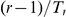 is the uniformly minimum variance unbiased estimator of 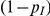 based on 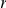 exponential random variables with unknown mean 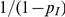. Furthermore, 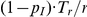 converges almost surely to 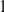, as 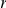 tends to infinity; in particular, the point predictors in part (i) and (ii) are strongly consistent.

We also note that the logarithm of the statistic in part (i) under-estimates 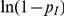 in average. In fact, the difference between the natural logarithm of the statistic in (i) and the statistic in (ii) is 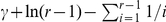, which is negative for 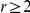, and increases to zero as 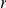 tends to infinity. From a computational stand point, however, the statistics 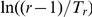 and 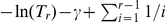 differ by at most 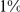-units when 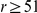. The same precision may be reached for smaller values of 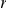 if larger bases are utilized. For instance, in base-10, the discrepancy will be at most 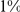 for 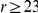.

In regards to part (iii) of the theorem, we note that our prediction intervals for 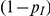 cannot contain zero unless 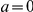. On the other hand, since the density function used in equation (5) is unimodal, the shortest prediction interval for 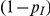 corresponds to a pair of non-negative constants 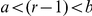 such that:
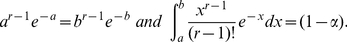
(6)Similarly, optimal prediction intervals for 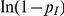 follow when
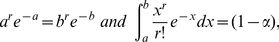
(7)with 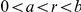 (see Materials and Methods for a numerical procedure to approximate these constants). In either case, because 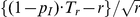 converges in distribution to a standard Normal as 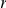 tends to infinity, one may select in (5) the approximate constants 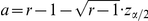 and 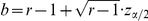. With these approximate values, if 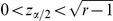 then the true confidence 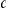 of the associated prediction intervals satisfies (see Materials and Methods):
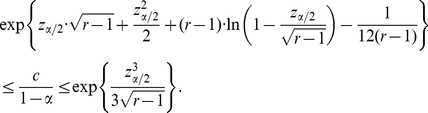
(8)(The term on the exponential on the left-hand side above is big-O of 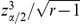; in particular, the lower-bound is of the same asymptotic order than the upper-bound.) We note that the constants produced by the Normal approximation may be crude for relatively large values of 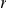, as seen in Table 2.

**Table 2:** Optimal versus asymptotic 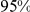 prediction intervals.

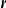	Predictioninterval for	Optimalconstants	Gaussianapproximation	Relativeerror 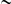
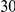	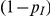	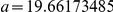 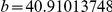	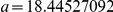 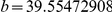	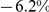 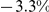
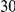	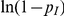	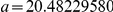 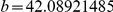	Same asabove	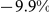 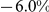
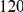	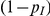	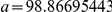 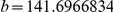	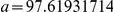 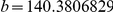	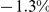 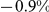
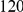	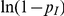	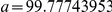 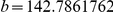	Same asabove	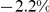 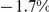

As high-throughput technologies allow deeper sampling of microbial communities, it will be increasingly important to have upper- and lower-bounds for 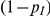 of a comparable order of magnitude. Since the prediction intervals for this quantity in Theorem 2 are of the form 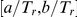, and the ratio between the upper- and lower-bound of this interval is 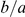, one may wish to determine constants 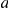 and 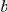 such that, not only (5) is satisfied, but also
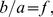
(9)where 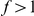 is a user-defined parameter. Not all values of 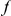 are attainable for a given 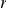 and confidence level. In fact, the smallest attainable value is given by the constants associated with the optimal prediction interval for 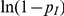. Equivalently, 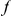 is attainable if and only if
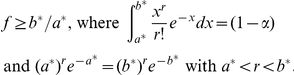
Conversely, and as stated in the following result, any value of 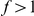 is attainable at a given confidence level, provided that the parameter 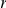 is selected sufficiently large.

**Theorem 3** *Let* 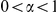 *and* 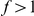 *be fixed constants. For each* 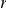 *sufficiently large, there are constants* 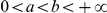 *such that (5) and (9) are satisfied.*

For a given parameter 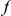, there are at most two constants 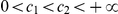 such that 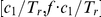 and 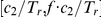 are prediction intervals for 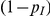 with exact confidence 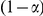. We refer to these as *conservative-lower* and *conservative-upper prediction intervals*, respectively. We refer to intervals of the form 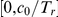 and 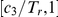 as *upper-* and *lower-bound prediction intervals*, respectively. See Table 3 for the determination of these constants for various values of 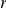 when 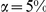.

**Table 3:** Constants associated with 95% prediction intervals.

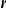	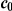	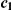	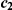	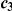
1	2.995732274	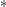	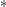	0.051293294
2	4.743864518	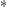	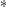	0.355361510
3	6.295793622	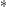	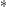	0.817691447
4	7.753656528	0.806026244	1.360288674	1.366318397
5	9.153519027	0.924031159	1.969902541	1.970149568
6	10.51303491	1.053998892	2.61300725	2.613014744
7	11.84239565	1.185086999	3.28531552	3.285315692
8	13.14811380	1.315076338	3.98082278	3.980822786
9	14.43464972	1.443547021	4.69522754	4.695227540
10	15.70521642	1.570546801	5.42540570	5.425405697
11	16.96221924	1.696229569	6.16900729	6.169007289
12	18.20751425	1.820753729	6.92421252	6.924212514
13	19.44256933	1.944257623	7.68957829	7.689578292
14	20.66856908	2.066857113	8.46393752	8.463937522
15	21.88648591	2.188648652	9.24633050	9.246330491
16	23.09712976	2.309712994	10.03595673	10.03595673
17	24.30118368	2.430118373	10.83214036	10.83214036
18	25.49923008	2.549923010	11.63430451	11.63430451
19	26.69177031	2.669177032	12.44195219	12.44195219
20	27.87923964	2.787923964	13.25465160	13.25465160
21	29.06201884	2.906201884	14.07202475	14.07202475
22	30.24044329	3.024044329	14.89373854	14.89373854
23	31.41481021	3.141481021	15.71949763	15.71949763
24	32.58538445	3.258538445	16.54903872	16.54903871
25	33.75240327	3.375240328	17.38212584	17.38212584

Constants associated with 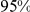 upper-bound, conservative-lower, conservative-upper and lower-bound prediction intervals for 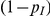, when 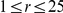 and 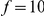. By definition, this means that 
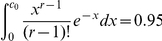
 and 
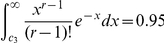
. Furthermore, the constants 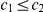 are solutions to the equation:
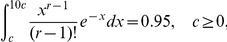
solved numerically with Newton's method using Maple 13.02. This equation may have at most two different solutions, and star (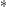) denotes that the equation has no solution.

### Effect of non-randomized sample sizes

The Embedding algorithm provides conditionally unbiased predictors and intervals for 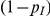 and 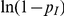, provided that an arbitrary number of additional observations is possible until observing 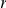 balls with colors outside 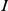. When dealing with fixed sample sizes, there is a positive probability of not meeting this condition, in which case the Embedding Algorithm is inconclusive. In large samples however, such as those collected in microbial datasets, the algorithm may be applied sequentially until it yields an inconclusive prediction. In such case, the true confidence of the prediction intervals produced by the algorithm satisfy the following.

**Theorem 4** *Suppose that condition (5) is satisfied. Conditioned on* 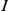 *, if* 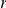 *balls with colors outside* 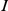 *are observed in the next* 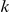 *draws from the urn, then the true confidence* 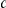 *of the prediction interval for* 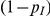 *produced by the Embedding algorithm satisfies:*

- *if* 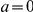 *then* 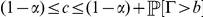 **;
- *if* 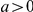 *then* 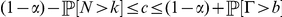, *where* 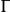 *is a Gamma random variable with parameters* 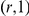, *and* 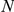 *is a Negative Binomial random variable with parameters* 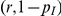.

Thus, if the Embedding algorithm produces an output in what remains of a finite sample size, the upper-bound prediction interval for 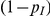 has at least the user-defined confidence. This is perhaps the case of most interest in applications: it allows the user to estimate the least number of additional samples to observe a color not seen in any sample. For the other three interval types, the true confidence is approximately at least the targeted one if the probability that the algorithm produces an output in what remains of the sample is large.

## Discussion

### Comparisons with Robbins-Starr estimators

Note that, like Robbins' and Starr's estimators, our method requires extracting additional balls from the urn to make a prediction. However, unlike the methods of the Introduction, our method uses only the additionally collected data–instead of all the data ever collected from the urn–to make a prediction. In terms of sequential analysis, this is advantageous to recover from earlier erroneous predictions (we expand on this point in the next section, see Fig. 1).

In what remains of this section, 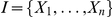 hence 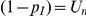, the conditional uncovered probability of a sample of size 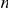. Furthermore, to rule out trivial cases, we assume that 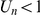 with positive probability i.e. the urn is composed by more than just balls of a single color.

Part (i) of Theorem 1 provides a conditionally unbiased predictor for 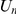. We can show, however, that Robbins' and Starr's estimators are not conditionally unbiased for 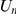 in the non-parametric case when 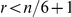. To see this argument, first notice that 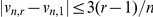 due to the inequality (3). On the other hand, if 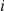 is a color in the urn such that 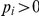 then
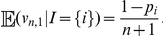
As a result:
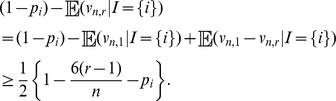
Hence, if there exists a color 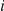 in the urn that makes the above quantity strictly positive (there are infinitely many such urns, including all urns composed by infinitely many colors, because 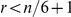) then 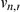 cannot be conditionally unbiased for 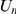.

On the other hand, due to parts (i) and (ii) in Theorem 1, we obtain (see Materials and Methods):
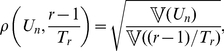
(10)

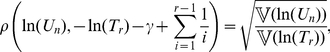
(11)where 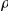 denotes correlation and 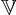 variance. Consequently, the point predictors in Theorem 1 are positively correlated with the quantities they were designed to predict. This contrasts with Robbins' estimator, which may be strongly negatively correlated with 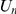. For instance, if 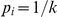 for 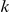 different colors in the urn, it is shown in [21] that the asymptotic correlation between 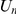 and Robbins' estimator 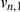 is asymptotically negative when 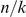 converges to a strictly positive but finite constant 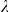. In this same regime but provided that 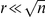, we can show that (see Materials and Methods):
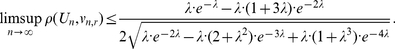
(12)Since the right-hand side above is negative for all 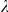 sufficiently small, Starr's estimator 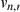 may also have a strong negative correlation with 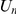 when 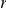 is much smaller than 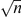.

A further calculation based on parts (i) and (ii) in Theorem 1 shows that
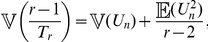


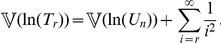
In particular, for fixed 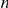, the correlations in equations (10) and (11) approach to one as 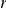 tends to infinity.

Finally, for non-trivial urns with finite 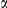-diversity, i.e. urns composed by balls with at least two but a finite number of different colors, one can show for fixed 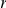 that the correlation in equation (10) approaches 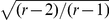 as 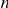 tends to infinity. Furthermore, if we again assume that 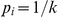 for 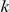 different colors in the urn and 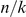 converges to a strictly positive but finite constant, then the correlation in equation (10) approaches zero from above. As we pointed out before, in this regime, Robbins' estimator is asymptotically negatively correlated with 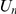.

### Selection of parameters

There are two main criteria to select the parameter 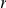 of the Embedding algorithm in a concrete application.

One criteria applies for point predictors. In this case, conditioned on 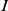, the standard deviation of the relative error of our prediction of 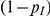 is 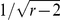 (Theorem 2, part (i)). To predict 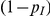, 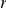 should be therefore selected as small as possible so as to meet the user's tolerance on the average relative error of our predictions. The same criteria applies for point predictors of 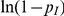, for which the standard deviation of the absolute error is of order 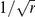, uniformly for all 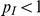 (Theorem 2, part (ii)).

A different criteria applies for prediction intervals. In this case, conditioned on 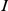, the user should first specify the confidence level, and how much larger he wants the upper-prediction-bound to be in relation to the lower-prediction bound of 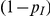. Since the ratio between these last two quantities is given by the parameter 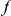 in (9), 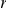 should be selected as small as possible to meet the user's pre-specified factor 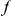 for the given confidence level of the prediction interval (Theorem 3). See Table 4 for the optimal choice of 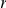 for various values of 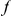 when 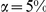. Note that for the selected parameter 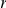, the constants associated with the optimal prediction intervals are given in equations (6) and (7), see Materials and Methods.

**Table 4:** Optimal selection of parameter 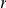 in terms of parameter 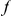.

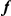	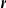	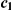	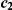
80	2	0.0598276655	0.355361510
48	2	0.1013728884	0.355358676
40	2	0.1231379857	0.355320458
24	2	0.226833483	0.346045204
20	3	0.320984257	0.817610455
12	3	0.590243030	0.787721610
10	4	0.806026244	1.360288674
6	6	1.822307383	2.58658608
5	7	2.48303930	3.22806682
3	14	7.17185045	8.27008349
2.5	19	11.26109001	11.96814857
1.5	94	75.9077267	76.5492088
1.25	309	275.661191	275.949782

Constants associated with the controlled upper- to lower-bound ratio prediction intervals for 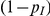, when 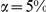; in particular, for each 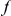 and 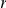, 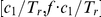 and 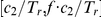 contain 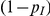 with a 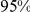 probability. For each 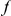, the smallest value of 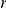 for which the equation:
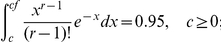
admits a solution, is reported. Numerical values where determined using Maple 13.02.

### Simulations on analytic and non-analytic urns

**Figure 4:**
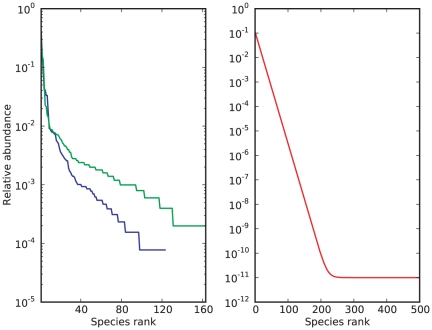
Rank curves associated with the human-gut, human-hand and exponential urn. In a rank curve, the relative abundance of a species is plotted against its sorted rank amongst all species, allowing for a quick overview of the evenness of a community. On the left, rank curves associated with the human-gut (blue) and -hand data (green) show a relatively small number of species with an abundance greater than 1%, and a long tail of relatively rare species. The right rank curve of the exponential urn (red) simulates an extreme environment, where relatively excessive sampling is unlikely to exhaust the pool of rare species.

We tested our methods against an urn with an exponential relative abundance rank curve over 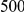 species, and an urn matching the observed distribution of microbes in a human-gut sample from [29]. We also analyzed a sample from a human-hand microbiota found in [30]. The gut and hand data are part of the largest microbial datasets collected thus far (see Fig. 4 for the relative abundance rank curve associated with each urn). The relative abundance rank curve, or for simplicity “rank curve”, associated with an urn is a graphical representation of its composition: the height of the graph above a non-negative integer 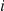 is the fraction of balls in the urn with the 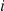-th most dominant color.

The blue dots and red curves on the plots on the left side in Fig. 1 show very accurate point predictions in log-scale of the conditional uncovered probability (as a function of the number of observations), when we apply the Embedding algorithm to a sample of size 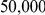 from the human-gut and exponential urn, respectively. In both instances, the parameter 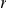 of the Embedding algorithm was set to 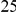. The accuracy of our method is confirmed by the red clouds on the plots on the right side of Fig. 1, which are centered around 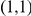. The red clouds also indicate that our predictions recover more easily from offset predictions as compared to Robbins' and Starr's (correlation coefficient of red clouds, 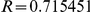 and 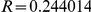 on top- and bottom-right, respectively). This is to be expected because the Embedding algorithm relies only on the additionally collected data to make a new prediction, whereas Robbins' and Starr's estimators use all the data ever collected from the urn. On the other hand, the red and blue curves in Fig. 2 show that the conservative-upper and -lower prediction intervals of the conditional uncovered probability (also as a function of the number of observations) contain this quantity with high probability and, unlike Esty's intervals, have a constant length in logarithmic scale. The intervals on the plots on the right side are tighter than those on the left because of the decrease of the parameter 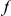 from 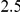 to 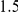. In each case, the parameter 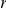 was selected according to the guidelines in Table 4. We note that sequential predictions based on the Embedding Algorithm in figures 1 and 2 were produced until the algorithm yielded inconclusive predictions. For this reason, our predictions ended before exhausting each sample.

In the human-hand dataset, 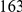 species were observed in a sample of size 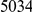. To simulate draws with replacement from this environment, we produced a random permutation of the data (see Materials and Methods section). Using the Embedding algorithm with parameters 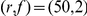, and according to our point predictor, 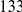 of the species observed in the sample represent 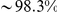 of that hand environment; in particular, the remaining 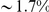 is composed by at least 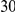 species. Furthermore, according to our upper-bound prediction interval, and with at least a 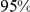 confidence, the species not represented in the sample account for less than 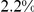 of that environment.

**Figure 5:**
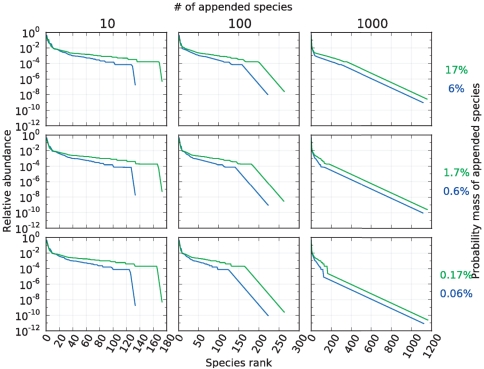
Rank curves associated with the rare biosphere simulation in the human-gut and -hand urn. Rank curves associated with Fig. 6 (green) and Fig. 7 (blue).

**Figure 6:**
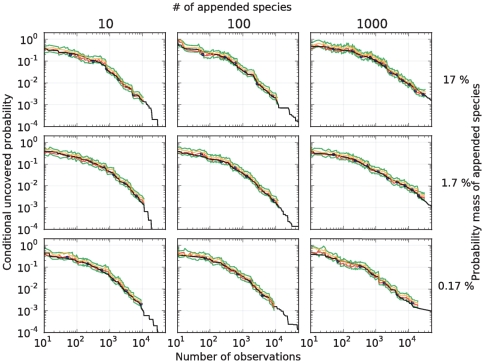
Predictions in the human-hand urn when simulating the rare biosphere. Prediction of the conditional uncovered probability (black) in nine urns associated with a human-hand urn. Point predictions produced by the Embedding algorithm (blue), point predictions produced by the algorithm each time a new species was discovered (red), 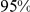 upper-bound interval (orange), and 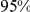 conservative-upper interval (green). The algorithm used the parameters 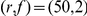. The different urns were devised as follows. For each 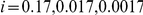 (indexing rows) and 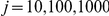 (indexing columns), a mixture of two urns was considered: an urn with the same distribution as the microbes found in a sample from a human-hand and weighted by the factor 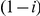, and an urn consisting of 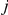 colors (disjoint from the hand urn), with an exponentially decaying rank curve and weighted by the factor 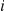. See Fig. 5 for the rank curve associated with each urn.

To test the above predictions, we simulated the rare biosphere as follows. We hypothesized that our point prediction of the conditional uncovered probability could be offset by up to one order of magnitude. We also hypothesized that the number of unseen species in the sample had an exponential relative abundance rank curve, composed either by 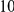, 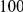 or 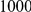 species. This leads to nine different urns in which to test our methods. These urns are devised such that they gradually change from the almost unchanged urn in the bottom left corner to the urn in the upper right, which is dominated by rare species (see Fig. 5 for the associated rank curves). As seen on the plots in Fig. 6, the Embedding algorithm yields very accurate predictions in each of these nine scenarios, for all the sample sizes considered.

**Figure 7:**
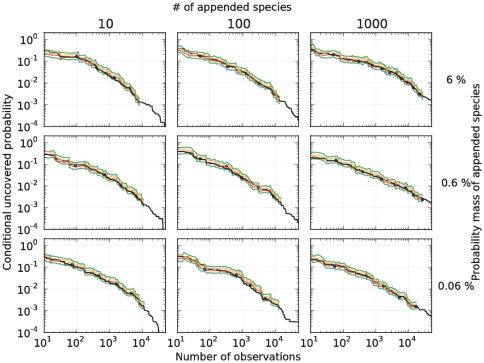
Predictions in the human-gut urn when simulating the rare biosphere. In a sample of size 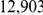 from a human-gut, 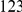 species were discovered. Based on our methods, we estimate that 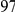 of these species represent 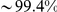 of that gut environment; hence, the remaining 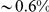 is composed by at least 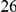 species. To test our predictions of the conditional uncovered probability (black), we simulated the rare biosphere by adding additional species and hypothesized that our point prediction could be offset by up to one order of magnitude: point predictions produced by the Embedding Algorithm (blue), point predictions produced by the algorithm each time a new species was discovered (red), 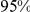 upper-bound (orange), and 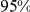 conservative-upper interval (green). The predictions used the parameters 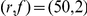. The different urns were devised as follows. For each 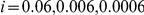 (indexing rows) and 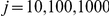 (indexing columns), a mixture of two urns was considered: an urn with the same distribution as the microbes found in the gut dataset, and weighted by the factor 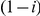, and an urn consisting of 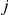 colors (disjoint from the gut urn), with an exponentially decaying rank curve and weighted by the factor 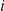. See Fig. 5 for the rank curve associated with each urn.

As seen in Fig. 7, our predictions are also in excellent agreement with the human-gut dataset when we simulate the rare biosphere. As expected, the conditional uncovered probability almost always lies between the predicted bounds. We also note that the predictions based on the Embedding algorithm are accurate even for a small number of observations. This suggests that our algorithm can be applied to deeply as well as shallowly sampled environments.

## Materials and Methods

### Heuristic behind the Embedding algorithm

The number of times a rare color occurs in a sample from an urn is approximately Poisson distributed. In the non-parametric setting, a direct use of this approximation is tricky because “rare” is relative to the sample size and the unknown urn composition. The embedding into a HPP is a way to accommodate for the Poisson approximation heuristic, without making additional assumptions on the urn's composition. To fix ideas, imagine that no ball in the urn is colored black. Make up a second urn with a single ball colored black. We refer to this as the “black-urn”. Now sample (with replacement) balls according to the following scheme: draw a ball from the original- versus black-urn with probability 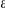 and 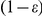, respectively, where 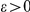 is a fixed but small parameter. Under this sampling scheme, even the most abundant colors in the original-urn are rare. In particular, the smaller 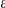 is, the closer is the distribution of the number of times a particular set of colors (excluding black) is observed to a Poisson distribution. This approach is not very practical, however, because the number of samples to observe a given number of balls from the original urn can be astronomically large when 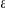 is very small. To overpass this issue imagine drawing a ball every 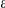-seconds. Draws from the original urn will then be apart 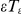 seconds, where 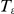 has a Geometric distribution with mean 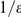. As a result: 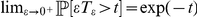, for 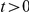. Thus, as 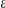 gets smaller, the time-separations between consecutive samples from the original urn resemble independent Exponential random variables with mean one. The black-urn can therefore be removed from the heuristic altogether by embedding samples from the original urn into a HPP with intensity one over the interval 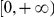.

### Simulating draws with replacement

To simulate draws with replacement using data already collected from an environment, produce a random permutation of the data. This can be accomplished with low-memory complexity using the discrete inverse transform method to simulate draws–without replacement–from a finite population [31].

### Constants associated with optimal prediction intervals

To numerically approximate a pair of constants 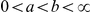 such that 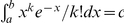 and 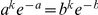, where the integer 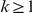 and the number 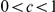 are given constants, introduce the auxiliary variable 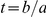, and note that the later condition is fulfilled only when 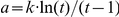 and 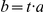. Due to Newton's method, the sequence 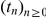 defined recursively as follows converges to the unique 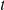 that satisfies the integrability condition, provided that 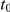 is chosen sufficiently close to 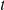:
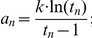


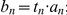


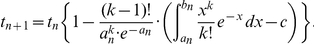



### Proof of Inequality (3)

First notice that
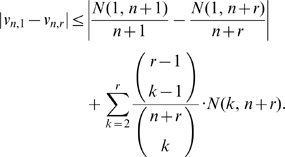
(13)


To bound the first term on the right-hand side above, notice that 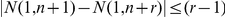. As a result, since 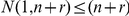, we obtain that:
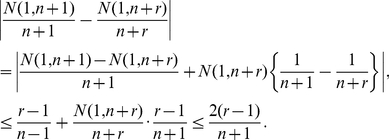
(14)


On the other hand, to bound the second term on the right-hand side of equation (13), define the quantity 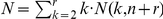 and notice that 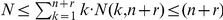. Using that a weighted average is at most the largest of the terms averaged, we obtain that:
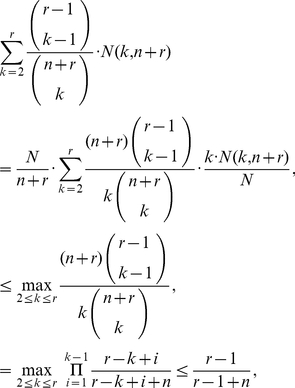
(15)where, for the last inequality, we have used that for each 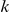, the associated product is less or equal to the factor associated with the index 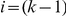. Equation (3) is now a direct consequence of equations (13), (14) and (15).

### Proof of Theorem 1

In what follows, 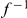 denotes the inverse function of 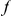.

Define 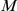 to be the set of decreasing partitions of 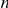 i.e. vectors of the form 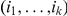, with 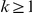 and 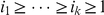 integers, such that 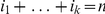. To each possible sample 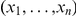, let 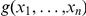 be the decreasing partition of 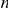 associated with the observed ranks in the sample.

Define 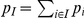, for each set 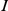 of colors. Part (i) in the theorem is equivalent to the existence of a function 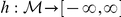 such that
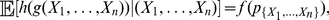
(16)with probability one. This is because, in the non-parametric setting, the different colors in the urn carry no intrinsic meaning apart from being different. If there is a certain function 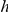 which satisfies condition (16) then 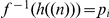, for each color 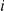 such that 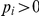. In particular, the set 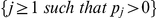 must be finite. Furthermore, if this set has cardinality 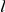 then 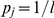, for each color 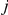 in the set; in particular, 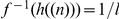. Condition (ii) is therefore necessary for condition (i). Conversely, if condition (ii) is satisfied and the urn is composed by 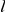 colors occurring in equal proportions then the function 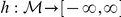 defined as 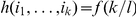 satisfies condition (16).

### Proof of Theorem 2

Conditioned on the set 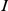, and the random index 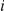 used in Step 3 of the Embedding algorithm, 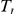 has a Gamma distribution with shape parameter 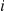 and scale parameter 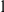. However, because 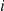 has a Negative Binomial distribution, conditioned on 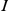 alone, 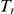 has Gamma distribution with shape parameter 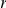 and scale parameter 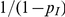. In particular, 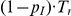 has probability density function 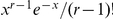, for 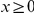. From this, parts (i) and (iii) in the theorem are immediate. To show part (ii), notice first that 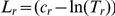 is conditionally unbiased for 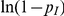, where
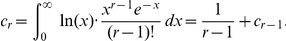
The second identity above is due to an integration by parts argument and only holds for 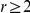. However, since 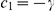, we obtain that 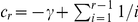, for 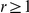. This shows that 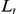 is conditionally unbiased for 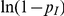. To complete the proof of the theorem, notice that 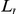 and 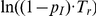 have the same variance. In particular, 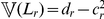, where
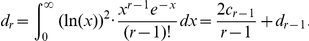
The last identity above holds only for 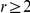. Using that 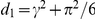, we conclude that 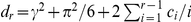, for 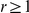. As a result: 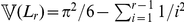; in particular, since 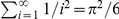, 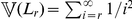. The theorem is now a consequence of the following inequalities:
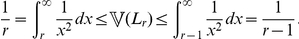



### Proof of Equation (8)

Let 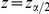 and assume that 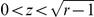. Observe that:
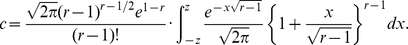
The factor multiplying the previous integral is an increasing function of 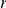; in particular, due to Stirling's formula, it is bounded by 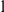 from above. Furthermore, from section 6.1.42 in [32], it follows that
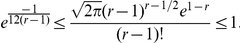
On the other hand, if one rewrites the integrand of the previous integral in an exponential-logarithmic form and uses that 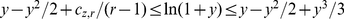, for all 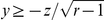, where
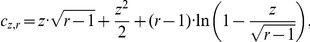
one sees that
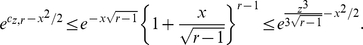
All together, these inequalities imply that
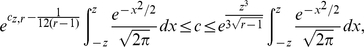
from which the result follows.

### Proof of Theorem 3

Due to the Central Limit Theorem, if 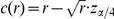 and 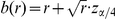 then
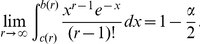
As a result, for all 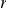 sufficiently large, 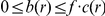, and the integral on the left-hand side above is greater than or equal to 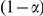. Fix any such 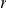. Since the value of the associated integral may be decreased continuously by increasing the parameter 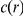, there is 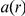 such that 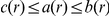 and
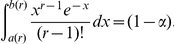
Define 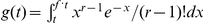, for 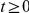. Since 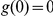 and, because 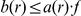, 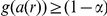, the continuity of 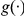 implies that there is 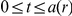 such that 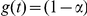. Selecting 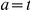 and 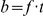 shows the theorem.

### Proof of Theorem 4

The proof is based on a coupling argument. First observe that one can define on the same probability space random variables 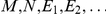 such that (1) 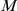 and 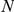 have Negative Binomial distributions with parameters 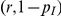, but with 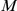 conditioned to be less than or equal to 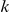; (2) 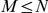 but 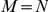 when 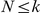; and (3) 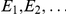 are independent Exponentials with mean 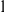 and independent of 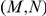.

Let 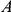 be the event “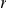 balls with colors outside 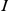 are observed in the next 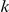 draws from the urn”. Conditioned on 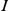, we have that 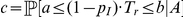 and 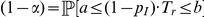. As a result:
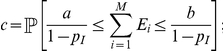


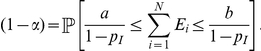
Since 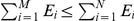, and because 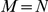 when 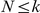, we obtain that
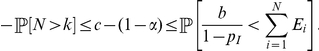
From this, the upper-bound in part (i) and both inequalities in part (ii) follow after noticing that 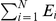 has a Gamma distribution with shape parameter 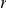 and scale parameter 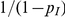. To show the lower-bound in (i), we again notice that 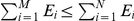. In particular, if 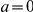 then
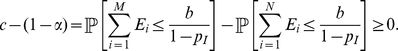



### Proof of Equations (10) and (11)

Consider random variables 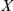 and 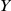 and a random vector 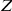, defined on a same probability space. Assume that 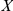 is square-integrable and conditionally unbiassed for 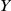 given 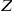 i.e. 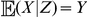. Furthermore, assume that 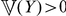 hence 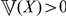. Because 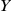 is also square-integrable and 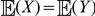, we obtain that
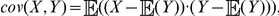


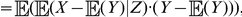


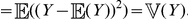
Hence 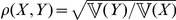.

Equation (10) follows by considering 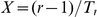, 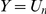 and 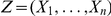. Similarly, equation (11) follows by considering 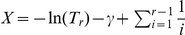 and 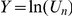.

### Proof of Inequality (12)

First note that
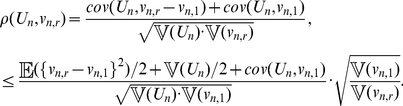
(17)Now observe that 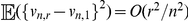 because of inequality (13), which implies that 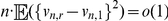 because 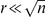. On the other hand, because Robbins' and Starr's estimators are both unbiased for 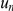, we have 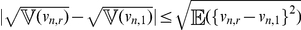. Furthermore, according to the proof of Theorem 2 in [21], 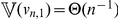, therefore
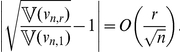
As a result, 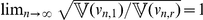. Inequality (12) is now a direct consequence of inequality (17), and the next identities [21]: